# The effect of periodontal therapy on neopterin and vascular cell adhesion molecule-1 levels in chronic periodontitis patients with and without acute myocardial infarction: a case-control study

**DOI:** 10.1590/1678-7757-2017-0199

**Published:** 2018-03-26

**Authors:** Zeynep Turgut Çankaya, Ayşen Bodur, Gülten Taçoy, Imge Ergüder, Derya Aktuna, Atiye Çengel

**Affiliations:** 1 Gazi University, Faculty of Dentistry, Department of Periodontology, Ankara, Turkey; 2 Gazi University, Faculty of Medicine, Department of Cardiology, Ankara, Turkey; 3 Gazi University, Faculty of Medicine, Department of Biochemistry, Ankara, Turkey; 4 Gazi University, Faculty of Medicine, Department of Biostatistics, Ankara, Turkey

**Keywords:** Periodontitis, Gingival crevicular fluid, Neopterin, Vascular cell adhesion molecule-1, Myocardial infarction

## Abstract

The presence of neopterin in gingival crevicular fluid (GCF) is a marker for local and acute immune activation, and the presence of vascular cell adhesion molecule (VCAM-1) in GCF is accepted as a marker for chronic vascular inflammation.

**Objectives:**

This study aimed to evaluate effects of periodontal treatment on GCF levels of neopterin and VCAM-1 in patients with chronic periodontitis (CP) with acute myocardial infarction (AMI) compared with systemically healthy CP patients.

**Material and methods:**

Sixty subjects (20 CP patients with AMI, 20 healthy CP patients, and 20 healthy controls) were included. GCF samples were analyzed at baseline and after 3 and 6 months, and the probing pocket depth (PD), clinical attachment level (CAL), bleeding on probing, gingival (GI) and plaque (PI) indices were recorded. We determined neopterin and VCAM-1 levels (concentration and total amount) using enzyme-linked immunosorbent assay (ELISA). No significant differences were seen between the AMI+CP and CP groups for PI, GI, GCF levels of neopterin and VCAM-1 at baseline.

**Results:**

The number of teeth with 5 mm≤CAL<7 mm and CAL≥7 mm were significantly increased in the AMI+CP group at baseline. There were no significant differences between the AMI+CP and CP for PI, CAL, GCF volumes, and the AMI+CP group had the highest clinical improvement in the number of teeth with 5 mm≤CAL<7 mm at the sixth month. There were significant positive correlations between clinical periodontal inflammation and the presence of neopterin and VCAM-1 in GCF prior to and following periodontal treatment, and between the GCF volume and clinical parameters.

**Conclusions:**

Data suggest that the total amount and concentration of neopterin and VCAM-1 in GCF seemed to be closely associated with periodontal disease severity in CP patients with AMI. Moreover, the results of our study demonstrate that the past periodontal status is potentially correlated between groups, with similar periodontal disease severity.

## Introduction

Periodontitis is a bacterially-induced, localized and chronic inflammatory disease that destroys the connective tissue and bone that support the teeth. Periodontitis and acute myocardial infarction (AMI) are two diseases that share common risk factors^12^. In recent years, literature has paid attention to positive correlation between periodontitis and coronary heart disease, acute coronary events, including AMI^4^. In these studies, periodontitis is defined by clinical examination or radiologic criteria^6^ ^,^ ^25^. Such trials are limited regarding gingival crevicular fluid (GCF)-based design and do not include periodontal treatment results. Site-directed measurements may allow for a more definitive identification of susceptible individuals and evaluation of responsiveness to therapy^10^.

Neopterin (N) is produced by interferon-gamma-stimulated monocytes. Elevated serum N concentrations predicted future adverse cardiac events in patients with angina pectoris^2^ and one investigation demonstrated a relationship with the extent of coronary atherosclerosis^30^. The determination of N in GCF might be useful for diagnosing and predicting periodontal diseases^21^. Studies evaluated the N status in various human biological fluids, including GCF^21^ ^,^ ^22^.

A number of cell adhesion molecules have been detected as soluble circulating forms in human serum and other body fluids^10^. Vascular cell adhesion molecule-1 (VCAM-1) is a member of the immunoglobulin superfamily of adhesion molecules and expressed on cytokine-activated endothelial cells. There are studies that investigated the levels of several adhesion molecules in different forms of periodontitis^24^ ^,^ ^26^. However, there are no studies specifically addressing the altered GCF profile concurrent with the onset of AMI (i.e. in the first 24 h) and no data are currently available about GCF N and VCAM-1 levels in AMI patients with chronic periodontitis (CP). Thus, in this study, we aimed at assessing whether GCF levels of CP patients with AMI have an alteration in GCF levels of N and VCAM-1. We also assessed whether these alterations might be related to treatment of existing periodontitis in AMI patients. We hypothesized that severe CP may play a role in initiating or exacerbating MI, and that there is an increased risk of AMI among systemically healthy people affected with severe CP. To test these hypotheses, we aimed to evaluate the effects of periodontal treatment on GCF levels of neopterin and VCAM-1 in CP with AMI in comparison to systematically healthy CP patients. We also assessed whether AMI was associated with a local inflammatory response, as reflected in the levels of GCF N and VCAM-1. In addition, we tested the hypothesis that long-term exposure to severe AMI+CP in patients contributes to the development of myocardial infarction (MI), using a case-control model of MI to study the inflammatory response in GCF. To do this, we compared AMI patients with CP with those who were systemically healthy and had CP.

## Material and Methods

The protocol of this clinical trial (ClinicalTrials.gov Identifier: NCT03005886) was approved by the Ethical Committee of the Faculty of Medicine. This study was executed between March 2006 and March 2010. Participants were recruited from individuals referred to the Department of Cardiology, Faculty of Medicine and Department of Periodontology, Faculty of Dentistry. Participants were classified as AMI+CP, CP and healthy control. Informed written consent was obtained from all subjects.

### Inclusion/exclusion criteria

Patients, who were referred to the Department of Cardiology and met the AMI diagnostic criteria^29^, with or without persistent ST-segment elevation, were screened for inclusion in the study. Patients with neoplasias, liver cirrhosis, HIV infection, chronic renal failure, hypo or hyperparathyroidism, diabetes mellitus and chronic inflammatory diseases were not included. None of the patients had received periodontal treatment during the past six months and none had received antibiotic medication during the past three months. AMI patients were regarded as suitable for the study if they were affected by CP and had at least 16 teeth, including at least four molars in different quadrants and at least two periodontal pockets at least 5 mm in depth, with a minimum of 2 mm attachment loss. Data for 140 AMI patients were obtained from patient files in the Department of Cardiology. Serum N and VCAM-1 levels of these patients were measured from samples that were collected at the time of their admission to the coronary intensive care unit. Sixty AMI patients, who had been stabilized for 24 to 48 h after infarction, volunteered for periodontal examination in the coronary care unit. Of these, 53 met the inclusion criteria, but only 20 of them were approved to be included in this study. All patients with AMI were on medical therapy, and none of them used antiaggregant therapy other than salicylates. A total of 85 CP patients without history of systemic conditions were examined from individuals referred to the Department of Periodontology, Faculty of Dentistry. Of these, 65 returned for blood sample collection, medical assessment and cardiology consultation at the Department of Cardiology. Finally, a group of 20 systemically healthy volunteer CP patients participated in the study. In addition, to the abovementioned controls, 20 clinically healthy individuals who did not have any periodontal and systemic disease history were included in the scope of this study. All control subjects were referred to a cardiology specialist in the same hospital clinic, who conducted a comprehensive medical examination, including electrocardiogram, to confirm that they did not have any cardiovascular disease (CVD).

### Clinical procedures

The periodontal examination included the assessment of plaque index (PI)^27^, gingival index (GI)^16^, probing depth (PD), bleeding on probing (BOP) and clinical attachment level (CAL). All subjects underwent a periodontal examination performed by the same periodontist (ZTÇ). Prior to the study, the examiner was calibrated for reproducibility of PD and CAL measurements. To determine repeatability of the PD and CAL measurements, six sites *per* tooth in ten patients, were measured twice. For PD 98% and for CAL 99% of the paired measurements were within ±1 mm. All periodontal parameters were measured with a Williams periodontal probe calibrated in millimeters (Nordent Manufacturing Inc., Elk Grove Village, IL, USA). Periodontal measurements were taken at six sites *per* tooth (mesio-buccal, mid-buccal, disto-buccal, mesio-palatal, mid-palatal and disto-palatal). The deepest six pockets found in the different segments of each subject were chosen for GCF sampling. Baseline periodontal examination of AMI patients and 24-48 h GCF collection were carried out in their hospital bed under sufficient illumination using artificial light. The examiners could not be “blinded” to the subject's general condition, since they were examined in a hospital. Within a time period of two months after the proceeding infarction, none of the patients had received periodontal treatment. AMI patients underwent periodontal therapy after the stabilization of their condition with the consent from same cardiologist. Periodontal disease was diagnosed based on the 1999 classification system developed by Armitage, and a preoperative periapical radiograph was taken, which provided baseline data in Faculty of Dentistry. All selected patients underwent a 2- to 4-week initial therapy, which included comprehensive proper plaque control program, scaling, subgingival curettage and root planning in Department of Periodontology. In all patients a periodontal reevaluation was performed four weeks after phase I therapy, to confirm the suitability of the sites for periodontal surgery. Mucoperiosteal flap operation was performed in cases when needed. Periodontal treatment including periodontal surgery was completed on the basis of 20 AMI and 20 CP patients, periodontal examination and GCF sampling repeated in baseline (T0), 3^rd^ (T3) and 6^th^ (T6) months.

### GCF sampling and processing

We collected GCF samples using commercially available periopaper (Oraflow Inc., Box 219 Plainview, NY), as described previously^20^. The sample site was gently air-dried and all supragingival plaque was removed. The area was carefully isolated with cotton rolls and a saliva ejector was used to prevent samples from being contaminated. Periopaper strips were inserted into the pockets until slight resistance was felt and left in place for 30 s. We tried to avoid mechanical injury of the gingival tissues. Strips contaminated by bleeding or exudate were discarded. The amount of GCF on the strips was measured by weighing the accumulated fluid. Strips were placed into Eppendorf tubes. Weighing was then repeated immediately after collection to overcome any evaporation and then stored at -80°C until processed.

### Laboratory analyses

Blood samples for serum were centrifuged for 10 min at 11.00 RPM separating serum from cells. Serum samples were then immediately divided into 0.2-0.5 mL aliquots and stored at -80°C until required for analysis. We assayed samples for N and VCAM-1 using quantitative enzyme immunoassays. Microcentrifuge tubes containing periopaper strips with absorbed GCF sample were allowed to reach room temperature and eluted using a centrifugal method^8^. After centrifugation, strips were removed and the fluid was assayed by ELISA for N and soluble VCAM-1. The levels of N and VCAM-1 in serum and GCF samples were measured using ELISA kit (Demeditec, Germany; Biosource, Invitrogen, Carlsbad, CA, USA). The ELISA plates were assessed spectrophotometrically at 450 nm. The concentrations of N and VCAM-1 in each sample were calculated by using the standards included with the kit. Results were expressed as ng/ml. Total amounts were also calculated by multiplying concentrations and GCF volumes^31^.

### Statistical analysis

We performed data analysis using the SPSS statistical package program (Microsoft Corp., Chicago, IL, USA). For categorical variables, we used the chi-square test to compare two or more groups, and for metric variables, the Mann-Whitney U test for two groups, and for more than two groups, the Kruskal-Wallis analysis of variance (ANOVA). Two-way repeated measures ANOVA were used to evaluate both within and between group comparisons. Spearman correlation coefficient was calculated for assessing the association between variables. Frequency (percentage) for categorical variables, mean±standard deviation, median (minimum-maximum) for metric variables were given as descriptive statistics. We considered p<0.05 as statistically significant. When the power analysis is made for CAL, the power is sound 0.993 for 20 sample in each group.

## Results

**Figure 1:**
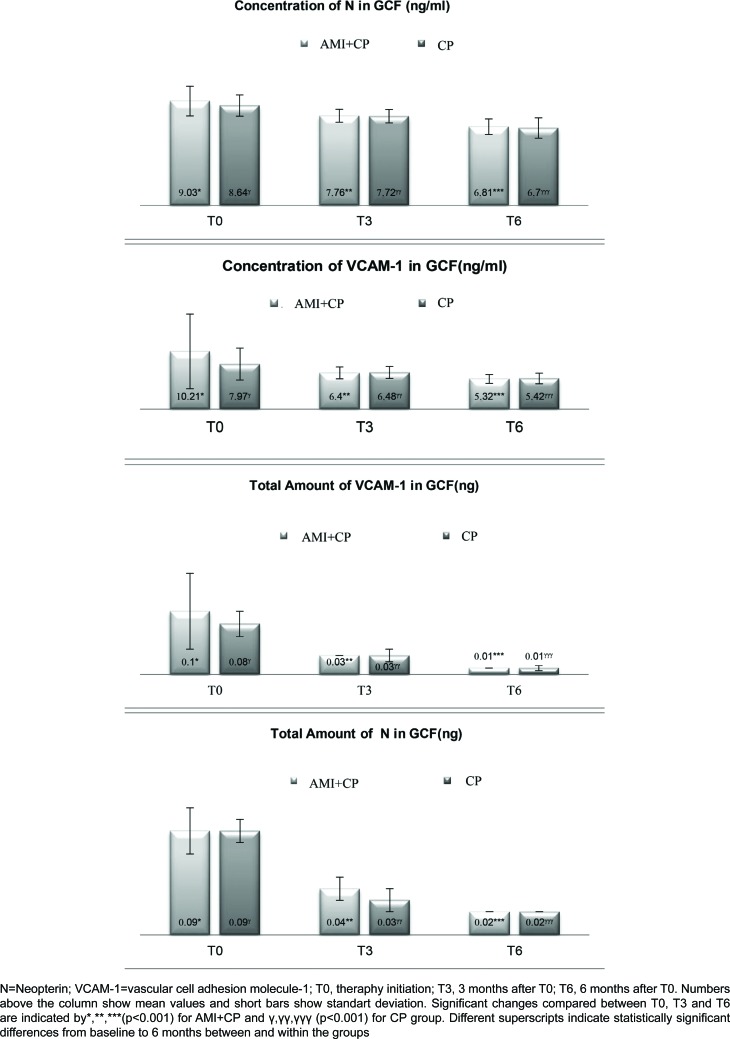
Concentration and total amount of N and sVCAM-1 in GCF of chronic periodontitis patients with and without AMI prior to and following therapy

In total, sixty subjects were enrolled. In this scope, forty CP patients with and without AMI were examined in the third and sixth months following the periodontal treatment. No significant differences were found among the three groups with regarding gender, education status, level of income, number of teeth, and serum N values at baseline (Table 1). The AMI+CP group had higher serum VCAM-1 levels compared with the other two groups. There were no significant differences between the CP and AMI+CP groups concerning PI and GI, which, in addition, were higher in both groups when compared with the healthy control group (Table 2). An analysis was carried out to investigate the relationship between periodontal disease severity and AMI using the number of teeth with a CAL degree of 5 mm or more (Table 3). In AMI patients, there were significantly fewer individuals with mild periodontitis and significantly more individuals with severe (and moderate-to-severe) periodontitis compared with patients in the CP group. At the third and sixth months, there were no significant differences between CP and CP+AMI groups for PI and CAL (Table 4). Within each group, all clinical parameters showed statistically significant decreases at the third and sixth months. The AMI+CP group showed statistically significant decreases in the number of teeth with 5≤CAL<7 and CAL≥7 mm after the periodontal treatment (Table 5). AMI+CP and CP groups showed significant decreases in total amounts and concentrations of N and VCAM-1 in the GCF during the post-treatment period (Figure 1). The decreases in the AMI+CP group were greater than those in the CP group, and this difference approached statistical significance (p<0.001). During pre- and post-treatment periods, there were no statistically significant differences between the two groups regarding the concentration and total amount of N and VCAM-1. There were significant positive correlations between concentrations, the total amount of GCF N and VCAM-1 and the periodontal variables at the given time points in the AMI+CP and CP group (Table 6). The concentration of VCAM-1 in GCF was negatively correlated with BOP in the AMI+CP group.

**Table 1:** Study subject characteristics

	AMI+CP	CP	Healthy	
	n=20	n=20	n=20	p
Age(years)	56.5±8.02*(42-74)	47.25±7.34(39-73)	49.1±5.3(39-58)	<0.001
BMI(kg/m^2^)	27.69±3.63**(23.29-35.16)	25.09±2.28(20.8-30.61)	24.1±2.15(20.31-29.41)	<0.01
Smoking(n-%)	17§-%85	6-%30	3-%15	<0.001
Education status	2-2-12-2-2	2-2-10-3-3	1-1-8-6-4	>0.05
Level of income	6-14	7-13	6-14	>0.05
Number of teeth	22.05±5.05(7-28)	22.65±3.03(16-28)	23.55±1.84(20-26)	>0.05
GCF volume(ml)	0.01±0.001(0.008-0.01)	0.01±0.001(0.008-0.01)	0.003±0.0006°(0.002-0.004)	<0.001
Serum N v(mg/ml)	20.81±4.54(16.08-34.50)	19±1.21(17.49-21.86)	19.06±2.41(16.65-23.47)	>0.05
GCF N c(ng/ml)	9.03±1.27(7.45-11.55)	8.64±0.94(6.84-11.31)	7.67±0.82°(5.67-8.84)	<0.001
GCF N ta(ng/ml)	0.09±0.02(0.07-0.16)	0.09±0.01(0.06-0.14)	0.02±0.00(0.01-0.03)	<0.001
Serum VCAM-1 (ng/ml)	1824.8±4597.9°(1230.9-3264)	1334.7±256.3(1017.0-2071)	1290.9±264.2(754.8-1938)	<0.01
GCF VCAM-1 c(ng/ml)	10.21±6.60(5.79-36.19)	7.96±2.82(4.94-16.81)	7.23±2.34°(3.95-13.79)	<0.05
GCF VCAM-1 ta(ng)	0.1±0.06°(0.06-0.33)	0.08±0.02°(0.04-0.14)	0.02±0.009°(0.01-0.04)	<0.001

Data given as mean±standard deviation(min-max)values. n=Number of patients. The education status data given as the number of participants in primary school, secondary school, high school, university and graduate. The level of income describes number of participants with good or medium income.

* Statistically significant difference between groups; Anova.

** Statistically significant difference between groups;Kruskal-Wallis test.

§ Statistically significant difference between groups; Chi-square test.

° Statistically significant difference between groups; Kruskal-Wallis test. BMI=body mass index, N=Neopterin. GCF=Gingival Crevicular Fluid. VCAM-1=Vascular cell adhesion molecule. c=concentration, ta=total amount.

**Table 2:** Baseline periodontal parameters

	AMI+CP	CP	Healthy	
	n=20	n=20	n=20	p
Pl(full-mouth)	1.83±0.49*	1.58±0.52	1.37±0.22*	<0.05
GI(full-mouth)	1.67±0.42	1.46±0.33	1.14±0.26¥	<0.001
PD(full-mouth)	3.02±0.73¥	3.54±0.77¥	2.05±0.19¥	<0.001
CAL(full-mouth)	3.71±0.89	5.13±1.07¥	0.00	<0.001
BOP(full-mouth)	0.29± 0.16	0.52± 0.22¥	0.23± 0.18	<0.001
PI(sampled-sites)	2.01±0.46	1.81±0.57	1.00±0.30¥	<0.001
GI(sampled-sites)	1.72±0.36	1.55±0.37	0.98±0.23¥	<0.001
PD(sampled-sites)	3.75±1.05¥	5.82±1.20¥	1.81±0.22¥	<0.001
CAL(sampled-sites)	4.41±1.12	6.55±1.12¥	0.00	<0.001
BOP(sampled-sites)	0.36±0.24¥	0.7±0.18¥	0.15±0.18¥	<0.001

Data given as mean±SD; p value by Kruskal Wallis Test for PI=plaque index; GI=gingival index; PD=probing depth; BOP=bleeding on probing; p value by Mann-Whitney Test for CAL=clinical attachment level.

* Statistically significant difference(p<0.05).

¥ Statistically significant difference(p<0.001)

**Table 3:** Number of teeth with a clinical attachment level (CAL) degree of 5 mm or more

	AMI+CP	CP	
Number of teeth with	n=20	n=20	p
CAL<5 mm	22.30±4.94(7-28)	22.35±2.85(16-28)	<0.001
5 mm≤CAL<7 mm	19.3±6.13*(6-17)	15.05±2.30(10-18)	>0.001
CAL≥7 mm	15.6±5.9*(1-22)	10.2±1.93(7-14)	<0.001

Data given as mean±SD(Min-Max)values; p value by Mann-Whitney Test.

**Table 4:** Clinical parameters of groups prior to and following periodontal treatment

	AMI+CP	CP	P	AMI+CP	CP	P
	n=20	n=20		n=20	n=20	
	Full-Mouth			Sample sites		
PI-T0	1.83±0.49^a^	1.58±0.52^b^	<0.001(a-b)	2.01±0.46^a^	1.81±0.57^b^	≥0.05
T3	1.5±0.4^c^	1.21±0.43^c^	≥005(c-c, d-d)	1.72±0.46^c^	1.44±0.50^c^	(a-a,b-b,c-c)
T6	1.1±0.4^d^	0.9±0.37^d^		1.34±0.42^d^	1.06±0.46^d^	
p	(a-c-d)<0.001	(b-c-d)<0.001		(a-c-d)<0.001	(b-c-d)<0.001	
GI-T0	1.67±0.42^a^	1.46±0.33^a^	≥0.05(a-a)	1.72±0.36^a^	0.97±0.15^a^	≥0.05
T3	1.3±0.4^b^	1.08±0.27^c^	<0.05(b-c-d)	1.32±0.34^b^	1.18±0.32^b^	(a-a,b-b,c-c)
T6	1.0±0.4^c^	0.76±0.24^d^		1.02±0.32^c^	0.85±0.29^c^	
p	(a-c-d)<0.001	(b-c-d)<0.001		(a-b-c)<0.001	(a-b-c)<0.001	
BOP-T0	0.29±0.03^a^	0.52±0.05^b^	<0.001	0.36±0.05^a^	0.70±0.04^b^	<0.001
T3	0.15±0.01^c^	0.32±0.03^e^	(a-b,c-e,f-d)	0.19±0.03^c^	0.42±0.03^e^	(a-b,c-e,f-d)
T6	0.08±0.00^d^	0.16±0.02^f^		0.09±0.01^d^	0.25±0.02^f^	
p	(a-c-d)<0.001	(b-e-f)<0.00^1^		(a-c-d)<0.001	(b-e-f)<0.001	
PD-T0	3.02±0.73^a^	3.54±0.77^b^	<0.001	3.75±1.05^a^	2.02±0.14^b^	<0.001
T3	2.5±0.6^c^	3.05±0.76^e^	(a-b,c-d,e-f)	3.30±1.03^c^	5.21±1.19^d^	(a-b,c-d,e-f)
T6	2.2±0.6^e^	2.75±0.71^f^		2.94±1.09^e^	4.73±1.17^f^	
p	(a-c-e)<0.001	(b-d-f)<0.00^1^		(a-c-e)<0.001	(b-d-f)<0.001	
CAL-T0	3.71±0.89^a^	5.13±1.07^b^	<0.001	4.41±0.25^a^	6.55±0.25^b^	<0.001
T3	3.2±0.8^c^	4.57±1.00^d^	(a-b,c-d,e-f)	3.2±0.8^c^	4.57±1.00^d^	(a-b,c-d,e-f)
T6	2.8±0.8^d^	4.11±1.01^f^		2.8±0.8^e^	4.11±1.01^f^	
p	(a-c-e)<0.001	(b-d-f)<0.001		(a-c-e)<0.001	(b-d-f)<0.001	

Data given as mean±SD; p value by two way ANOVA. Same superscripts indicate that there is no statistically significant difference (p≥0.05), different superscripts indicate that the difference is statistically significant (p<0.001, p<0.05) ;PI=Plaque Index; GI=Gingival Index; PD=Probing Depth; CAL=Clinical Attachment Level; BOP=Bleeding on Probing; SD=Standart Deviation. T0, theraphy initiation; T3, 3 months after T0; T6, 6 months after T0

**Table 5:** Number of teeth according to the clinical attachment level (CAL)

Groups	Time Interval	CAL<5mm	5mm≤CAL<7mm	CAL≥7mm	
AMI+CP	T0	22.3±4.94^a^	19.3±1.67^b^	15.6±5.9^δ^	p<0.05(a-b-δ)
n=20	T3	22.3±4.94^a^	16.9±1.04^c^	13.1±5.11^α^	p<0.05(a-c-α)
	T6	22.5±4.90^a^	13.9±0.99^d^	10.8±4.71^β^	p<0.05(a-d-β)
		p≥0.05	p<0.001(b-c-d)	p<0.001(δ-α-β)	
CP	T0	22.3±2.85^a^	15.0±0.51^e^	10.2±1.93°	p<0.05(a-e-°)
n=20	T3	22.3±2.85^a^	12.0±0.53^f^	7.45±1.87^¥^	p<0.05(a-f-¥)
	T6	22.5±2.85^a^	9.3±0.52^9^	5.6±1.1.66^€^	p<0.05(a-g-€)
		p≥0.05	p<0.001(e-f-g)	p<0.001(°-¥-β)	

Data given as mean±SD; p value by two way ANOVA. Same superscripts indicate that there is no statistically significant difference (p≥0.05), different superscripts indicate that the difference is statistically significant (p<0.001, p<0.05) CAL=Clinical Attachment Level; T0, therapy initiation; T3, 3 months after T0; T6, 6 months after T0.

**Table 6:** Correlation between total amount concentration of N, VCAM-1 and clinical parameters (r)

AMI+CP	GCF N Cont.(T6)	GCF VCAM-1 T.A(T6)		
Full-mouth PI(T6)	0.557*			
Sample PI(T6)	0.480*			
Full-mouth CAL(T6)	0.580**			
Full-mouth BOP(T6)		-0.454*		
**CP**	**GCF N Cont.(T0)**	**GCF N T.A (T3)**	**GCF N Cont.(T6)**	
Full-mouth CAL(T0)	0.452*			
CAL≥7(T0)	0.467*			
Sample PD(T3)		0.472*		
Full-mouth BOP(T6)			0.543*	
Full-mouth PI(T6)			0.536*	
Full-mouth PD(T6)			0.543*	
**CP**	**GCF VCAM-1 Cont.(T0)**	**GCF VCAM-1 T.A(T0)**	**GCF VCAM-1 Cont.(T3)**	**GCF VCAM-1 T.A(T3)**
Sample PD(T0)	0.563**	0.586**		
Sample CAL(T0)	0.470*	0.481*		
CAL≥7(T0)		0.461*		
Full-mouth GI(T3)			0.467*	
CAL<5(T3)				0.460*
5<CAL≤7(T3)				0.499*
CAL≥7(T3)			0.482*	0.529*

* p<0.05,

** p<0.01 T.A:Total Amount (ng), Cont:Concentration (ng/ml), AMI:Acute Myocardial Infarction, CP:Chronic Periodontitis, N:Neopterin, VCAM-1:vascular cell adhesion molecule-1, PI:Plaque Index, CAL:Clinical attachment level, BOP:Bleeding on probing, PD:Probing Depth (r)= Spearman correlation coefficient.

## Discussion

To our knowledge, there have been no studies evaluating the concentration and total amount of serum and GCF VCAM-1 and N in association with AMI patients with CP. This study provides the opportunity to examine the GCF profile of AMI patients with CP obtained in the first 24 to 48 h after MI that have not been modified by medical and periodontal treatment. This study achieved the following issues: (i) evaluated markers in GCF were simultaneously assessed; (ii) N and VCAM-1 levels in the GCF were assessed within the first 24-48 h after AMI, which allowed us to evaluate whether there was a local inflammatory burden in chronically infected patients; this was important because CP enhances the expression of cytokines that might contribute to an acute cardiac event; (iii) the levels of markers were assessed at three time points: before the initiation of periodontal therapy and twice after therapy (at 3 and 6 months), which provided data on sequential changes in the GCF with periodontal therapy; (iv) a correlation was established between the serological and biochemical markers of CVD with periodontal clinical variables before the initiation of and following the periodontal therapy; (v) standardization of the methods was used to evaluate the systemic and periodontal health conditions of the enrolled individuals; (vi) matching of participants between the groups was possible based on gender, education status, level of income, number of teeth, and serum N values. Because of the selection criteria of the study, matching between groups was not possible for age, smoking and BMI variables. After adjusting for age, BMI and smoking, most of the studied parameters such as periodontal parameters, total amount of N, concentration and total amount of VCAM-1 were found stable. Only the GCF N concentration was affected by BMI, PI was affected by smoking and the number of teeth with 5<CAL<7 was affected by BMI.

The measurement of clinical attachment loss and PD are indicators of previous periodontal disease rather than of present activity^21^. In this study, periodontal examination consisted of both clinical periodontal variables and a GCF assessment. This is the first study to profile N and VCAM-1 in the GCF of patients with recent history of AMI with CP and to investigate the effect of a comprehensive periodontal treatment on within the levels of these molecules in the same patient group. The GCF samples were taken from patients with AMI within the first 24-48 h of their arrival to the hospital in the scope of this study. This relatively short time interval was chosen to examine the initial effects of the MI process on the GCF profile. The 24-48 h GCF collection reflected the actual N and VCAM-1 profile and the local presence of N and VCAM-1 within the gingival environment at the time of AMI. Patients with AMI and CP had worse PI scores than patients with CP alone. PI scores represent a measure of the infectious burden associated with periodontal tissues^9^. However, the increase in PI scores in patients with AMI may be due to their stay in the intensive care unit. GI and BOP represent measures of the severity of the inflammatory burden within the gingival tissues^9^. Although it may be expected that patients with AMI might have worse GI and BOP scores, because of the high rate of anti-platelet drug administration in these patients, carrying out a periodontal examination within 24-48 h of the infarction, eliminated this issue. The levels of N and VCAM-1 were similar in patients with AMI and those with CP only. In light of these results, it can be noted that the past periodontal history was not significantly different between the groups, indicating similar periodontal disease awareness, extent and severity. This similarity existed in both CP patients and patients with AMI, and it appears to be a reflection of the host response to periodontitis that is reflected in both N and VCAM-1 levels of patients. Determination of crevicular fluid levels of markers related to AMI is important to explain the exact role of periodontitis-associated systemic inflammation in AMI.

In a previous study, to define a threshold for periodontitis at which the risk of AMI was greatest, we compared different cutoff levels of pathologic PDs between groups^28^. PD values≥4 mm were defined as indicative of periodontal pathology^28^. The presence of>50% of sites with PDs≥4 mm showed the highest discrepancy between the groups, although this parameter lost its significance after an adjustment for known risk factors^28^. In the study mentioned, radiographs were not available, and periodontitis was defined with clinical measurements. The clinical periodontal findings of our study indicate a positive association between moderate and severe periodontitis and AMI, which was reported previously^6^ ^,^ ^28^. In studies by Beck, et al.^3^ (2001) and Arbes, et al.^1^ (1999) to evaluate the extent of periodontal disease, the percentage of tooth sites with different CAL levels were investigated. In this study, the association between CP and AMI varied by CAL levels, with a positive association between the number of teeth with CAL≥5 mm. We found that the number of teeth with a CAL of 5 mm or more was a significant predisposing factor for AMI in patients with CP when compared with healthy CP patients. We noted a possible similar modified effect with an association among patients with AMI+CP and, more importantly, among those with CP but not AMI. The AMI+CP patients demonstrated significantly increased of the disease severity as assessed by the number of teeth with a CAL of ≥5 mm. The interaction between CAL and AMI indicates that the cumulative destructive effects of CP on the periodontal tissues, and clinical periodontal infection with bleeding on probing on the same tooth with attachment loss, may cause long-term systemic side effects low-grade periodontal infection as an independent or additional predisposing factor for future AMI after simultaneously considering several other recognized risk factors for AMI.

Periodontal disease is suggested to affect cytokine levels^13^. Studies suggest that periodontitis exerts its clinical effects via the systemic dissemination of locally produced mediators, such as CRP, IL-6, IL-1β and TNF-α^11^ ^,^ ^17^. We chose to study N because changes in its levels are systemic, unlike other inflammatory cytokines (the levels of which only change at local lesion sites), and peripheral serum level of N may help to determine the severity of coronary atherosclerosis^18^. VCAM-1 is observed in the remote myocardium of experimental models of AMI^15^, while other adhesion molecules that are constitutively expressed remain unchanged^19^. It would be interesting to examine GCF versus serum N and VCAM-1 levels during the MI process. In this study, we found that serum and GCF N levels were similar in AMI+CP and CP patients. These similarities have clinical importance for patients with apparently healthy non-MI+CP patients because determination of the N level may help when performing risk stratification of patients with coronary artery disease. Measuring N in GCF might be useful for diagnosing and predicting periodontal disease since it has been well documented that T cells, in addition to other inflammatory infiltrates, mediate the immunopathologic events in the periodontal disease^22^. Özmeriç, et al.^21^ (2002) found that N increased in parallel with the severity of inflammatory disease. VCAM-1 is expressed on cytokine-activated endothelial cells and its induction occurs within 6 h^10^. Evidence suggests that some of the cell trafficking to the periodontium is probably fulfilled by VCAM-1^1^ ^,^ ^14^. VCAM-1 have been used for risk assessment for future cardiovascular disease^23^.

Statistical significant reduction in pocket depth, PI, GI, BOP and gain in clinical attachment were found after 3 and 6 months of the follow-up concerning baseline in AMI patients. Total amounts and concentrations of N and VCAM-1 were markedly reduced following periodontal treatment in AMI patients. Periodontal treatment may improve efficiency of MI treatment by reducing the infectious load of the body associated with inflamed periodontium. Inflamed periodontium can contribute to the dynamics of inflammatory reaction in the body. Thus, the existence of inflamed periodontium is more important in patients with periodontitis after MI, compared to those without periodontal disease. We observed lower levels of N and VCAM-1 levels in both CP patients and the patients with AMI after periodontal treatment, which would represent an anti-inflammatory protective factor for future MI in these patients.

In this study, the statistically significant decrease of VCAM-1 levels following periodontal therapy may be interpreted as diminished endothelial cell activation. Serum and GCF levels of VCAM-1 in patients with AMI+CP were statistically higher than those in the healthy controls. These findings showed that periodontal disease generates an inflammatory response associated with endothelial dysfunction, expressed as elevated VCAM-1. The reduction in the GCF level of VCAM-1 suggests a possible need for the periodontal treatment in patients with MI, who have severe CP. N and VCAM-1 were much higher in periodontitis patients than in healthy subjects prior to periodontal treatment. We found that following periodontal treatment, total amounts and concentrations of N and VCAM-1 in the GCF significantly decreased. It has been suggested that total cytokine amounts in GCF might be more representative of disease status than concentrations^13^. Reduced levels of N and VCAM-1 in GCF after periodontal disease therapy could reduce the risk of myocardial infarction in systemically healthy patients with periodontal disease.

This study demonstrated a significant positive correlation between clinical periodontal parameters and the presence of N and VCAM-1 in GCF prior to and following periodontal treatment. Therefore, we might conclude that the severity of periodontal disease influenced the levels of N and VCAM-1. GCF levels of N and VCAM-1 might reflect chronic periodontal pathogenic burden and its contribution to the systemic inflammatory burden. In AMI + CP group, GCF N concentration was positively correlated with full-mouth PI, sample PI and full-mouth CAL, while the total amount of GCF VCAM-1 was negatively correlated with full-mouth BOP after treatment. The correlation between CAL and GCF N may reflect the potential significance of local inflammation for systemic inflammatory burden in patients with AMI and periodontitis. In CP patients, GCF N and VCAM-1 were correlated with number of teeth with CAL≥7 mm. These correlations indicate a relationship of N and VCAM-1 with periodontal breakdown and an association of these levels that relate to CAL degree and potentially reflect the level of periodontal health. These correlations in CP patients suggest that CF levels of N and VCAM-1 likely reflect a direct local contribution to the body regarding the severity of periodontal disease. Cumulative measures of past periodontal disease (CAL) and measures of ongoing inflammatory activity (PD, BOP) differed significantly in the AMI patients. These correlations may reflect the long-term exposure to severe periodontal disease in AMI+CP patients and appears to contribute to the development of MI. This study reported similar serological, clinical and immunological characteristics between CP patients with and without AMI. These findings might offer an etiological explanation for the causal relationship between periodontitis severity and acute cardiac events. We hypothesized that CVD could be triggered by systemic mechanisms in addition to local inflammatory factors, being chronic periodontal infection one of the possibilities to be considered^5^. Periodontitis is regarded as an infection in which putative periodontal pathogens trigger a chronic inflammatory and an immune response against periodontal structures^7^. As a source of systemic inflammatory burden, it is biologically plausible to consider periodontal disease severity as a putative risk/predisposing factor for AMI. Our findings suggest that reduced VCAM-1 levels following periodontal treatment may play a role in improving endothelial function. Determination of N levels helps to perform risk stratification of systemically healthy CP patients. Periodontal disease therapy is potentially beneficial in post-AMI+CP patients and might play a preventive role in non-AMI+CP patients.

## Conclusion

In accordance with the outcomes of this study, we conclude that development of a protective model (including periodontal treatment needs) would provide greater public benefit than a single risk prediction regarding multiple behavioral risk factors. The findings of this study might be considered when determining the risk of AMI in individuals who have severe periodontal disease. The presence of CP in patients with AMI might form a vicious circle, mutually enhancing each other's severity in susceptible hosts. Further comprehensive studies are needed to investigate the independent role of severe periodontitis in triggering acute cardiac events and the possibility of a relationship between periodontitis and recurrent episodes of MI. This study highlights the possibility regarding periodontal disease therapy to be depended of changes in biological potentials of traditional factors through increase of their risk effect.
